# Frequency of TGF-***β*** and IFN-***γ*** Genotype as Risk Factors for Acute Kidney Injury and Death in Intensive Care Unit Patients

**DOI:** 10.1155/2014/904730

**Published:** 2014-07-23

**Authors:** Caren Cristina Grabulosa, Marcelo Costa Batista, Miguel Cendoroglo, Beata Marie Redublo Quinto, Roberto Narciso, Julio Cesar Monte, Marcelino Durão, Luiz Vicente Rizzo, Oscar Fernando Pavão Santos, Maria Aparecida Dalboni

**Affiliations:** ^1^Nephrology Division, Universidade Federal de São Paulo/UNIFESP, São Paulo, Brazil; ^2^Hospital Israelita Albert Einstein, São Paulo, Brazil; ^3^Tufts-New England Medical Center, Boston, MA, USA; ^4^Universidade Nove de Julho/UNINOVE, São Paulo, Brazil

## Abstract

Genetic variations in TGF-*β* and IFN-*γ* may interfere with proinflammatory cytokine production and, consequently, may be involved with inflammatory diseases, as acute kidney injury (AKI). We considered that genetic polymorphisms of these cytokines may have a crucial role in the outcome of critically ill patients. To investigate whether the genetic polymorphisms of rs1800470 (codon 10 T/C), rs1800471 (codon 25 C/G) from the TGF-*β*, and rs2430561 (+874 T/A) from IFN-*γ* may be a risk factor for ICU patients to the development of AKI and/or death. In a prospective nested case-control study, were included 139 ICU patients who developed AKI, 164 ICU patients without AKI, and 244 healthy individuals. We observed a higher frequency to T/A genotype for IFN-*γ* (intermediate producer phenotype) and higher frequency of TT GG and TC GG genotype (high producer) for TGF-*β* polymorphism in overall population. However, these polymorphisms have not been shown as a predictor of risk for AKI and death. We found an increased prevalence of high and intermediate producer phenotypes from TGF-*β* and IFN-*γ*, respectively, in patients in ICU setting. However, the studied genetic polymorphism of the TGF-*β* and IFN-*γ* was not associated as a risk factor for AKI or death in our population.

## 1. Introduction

Critically ill patients in the intensive care unit (ICU) have a high incidence of acute kidney injury (AKI) [1, 2] and consequently high mortality rates. AKI occurrence is a consequence of the pathophysiology complex of systemic inflammatory response syndrome (SIRS). In SIRS there is the activation of leukocytes, which produce oxidative stress, high adhesion molecules expression, and high serum levels of proinflammatory cytokines as TNF-*α* and IL-6. These cytokines have been associated with poor outcome [3, 4] and some inflammatory mediators have also been reported to contribute to the renal vasculature injury and may consequent development the AKI [5].

IFN-*γ* is a proinflammatory cytokine. It has been reported that ischemic kidney injury triggers production of several cytokines, such as TGF-*β*, IFN-*γ*, IL-6, TNF-*α*, and IL-1*β* in the inflamed kidney [6–9].

Roedder et al. have been reported in a renal transplant model that lower IFN-*γ* gene expression causes less infiltration of leukocytes and allograft rejection, demonstrating that this cytokine is associated with inflammation process and kidney injury [10].

TGF-*β*, another inflammatory cytokine, has been reported to induce chemotaxis for neutrophils, T cells, monocytes, and fibroblasts to the site of the injury. Thus, this biologic effect of TGF-*β*1 suggests that this cytokine may also contribute to inflammation and a central role in chronic kidney fibrosis [11–14].

In fact, some authors observed that increased TGF-*β* activity* in vivo* results in glomerular extracellular matrix accumulation, proteinuria, tubule-interstitial fibrosis, and the onset of chronic renal failure, suggesting that its activity in promoting the synthesis of extracellular matrix plays a crucial role in fibrotic deposition and the decline in renal function [15, 16].

Kopp et al. also related that increased levels of circulating TGF-*β*1 induced progressive renal disease characterized by mesangial expansion, accumulation of glomerular immune deposits and matrix proteins, and interstitial fibrosis in this transgenic mouse model suggesting that chronically elevated circulating levels of TGF-*β*1 induce progressive glomerulosclerosis [15]. Therefore, it appears that renal diseases may also result from the inflammation and from inappropriate regulation of TGF-*β* expression [17]. However, until this moment there is no data about the role of IFN-*γ* and TGF-*β* in critically ill patients as risk markers to AKI. Recently, the study of polymorphisms of genes involved in host immune response, including cytokines and other modulators of the inflammatory response, has been a subject of interest, since these genetic markers may be potential determinants of susceptibility to or severity of acute events such as AKI [18, 19]. The reason why some patients with SIRS develop AKI and others do not is still unclear. So, it is possible that polymorphisms of inflammatory cytokines genes, in particular IFN-*γ* and TGF-*β*, may be involved in the pathophysiology of AKI in critically ill patients.

Thereby, the aim of this study was to investigate the frequency of rs1800470 (codon 10 T/C), rs1800471 (codon 25 C/G) from the TGF-*β*, and rs2430561 (+874 T/A) from IFN-*γ* polymorphisms genotypes in critically ill ICU patients and to analyze the possible impact of these polymorphisms on AKI and death.

## 2. Materials and Methods

### 2.1. Subjects

This study was a prospective, nested case-control which included three hundred and three patients (*n* = 303) from Hospital Israelita Albert Einstein, Sao Paulo, Brazil, who had been in the ICU for at least 48 hours ago and two hundred forty-four (*n* = 244) healthy individuals from Hypertension and Cardiovascular Metabolism Center, UNIFESP, Sao Paulo, Brazil, were included (Control group). Overall individuals were over 18 years old. Of the 303 ICU patients, 139 patients developed AKI (AKI group) and 164 patients did not (No AKI group). AKI was confirmed by the Acute Kidney Injury Network** (**AKIN) and Risk, Injury, Failure, Lesion Renal and End-stage-Renal Disease (RIFLE) [20] criteria defined as an increase of three times of serum creatinine compared with a baseline serum levels of creatinine or the creatinine clearance <60 mL/min/mm^3^ that was calculated by Levey equation (MDRD—Modification of Diet in Renal Disease) according to K/DOQI [21].

Patients who developed AKI were matched for age and gender to the No AKI and Control group. The criteria of exclusion at moment of selection of patients were patients who are not considered for resuscitation, kidney transplant patients, patients in dialysis treatment (acute or chronic), and patients who had previously participated in this study.

This study was approved by the Research Ethics Committee of the Universidade Federal de São Paulo (UNIFESP), reference number: CEP 1520/05, and by the Ethics Committee of the Hospital Israelita Albert Einstein, reference number 06/381.

### 2.2. Materials

30 mL of blood in ethylene diamine tetra acetic acid anticoagulant (EDTA) was collected from each individual for renal function analysis (urea and creatinine, Labtest, Lagoa Santa, MG, Brasil and Jaffe modified method [22], resp.), lipid profile (cholesterol, HDL and LDL cholesterol, automated method equipment Cell-Dyn Ruby Abbott Diagnostics), and markers of SIRS (CRP and albumin, Immulite 1000 immunoassay system, IL, USA, and colorimetric method in automated equipment Olympus AU400, Dallas, TX, USA, resp.) and DNA extraction was performed in order to study the polymorphisms of TGF-*β* and IFN-*γ* (Genotyping tray, One Lambda, Inc., 21001 Kittridge, St, Canoga Park, CA, USA).

#### 2.2.1. Analysis of Codons 10 and 25 of TGF-*β* and +874 IFN-*γ* Polymorphisms

Genomic DNA was prepared from peripheral blood using standard techniques and was extracted* by* the DTAB/CTAB method [23]. The polymerase chain reaction (PCR) sequence specific primer (SSP) technique was used to analyze two SNPs.

The polymorphisms may result from the substitution of a single nucleotide (single nucleotide polymorphisms or SNPs). Most polymorphism of genes of cytokines are of the type microsatellite and SNP and they are located in the promoter region, the introns and untranslated regions (UTR of untranslated region). Polymorphisms located in introns of genes can have significant effects on transcription that may be related to changes in the structure of binding sites of promoters or facilitators structure segments (“enhancers”) or blocking (“silencers”) within introns. Consequently, the polymorphism may alter the rate of transduction, influencing the amount of protein produced by the cell [24].

The detection of genotypes was performed on the purified DNA using a commercially available cytokine genotyping tray (One Lambda, Inc., 21001 Kittridge St, Canoga Park, CA, USA). The bands were separated by 2% agarose gel electrophoresis, visualized under ultraviolet, and photographed using a gel electrophoresis image documentation system (*Kodak Digital Science Electrophoresis Documentation and Analysis System 120*). Quality checks to ensure correctness of the genotypes were carried out by independent rating of the results by two investigators. Patients were grouped into the predicted high, intermediate, or low producer phenotypes according to their genotypes as defined previously [25]: for TGF-*β* phenotypes were classified as high producers (TT GG and TC GG genotypes), intermediate producers (TC GC, CC GG, and TT GC genotypes), and low producers (CC GC, CC CC, TT CC, and TC CC genotypes). Of the possible nine combinations of codon 10 and codon 25 genotypes, only one genotype was not present (TC/CC) in these study groups.

IFN-*γ* phenotypes were classified as high producers (TT genotype), intermediate producers (TA genotype), and low producers (AA genotype).

#### 2.2.2. Statistical Analysis

Data were analyzed using the statistical software SPSS (version 20.0, Chicago, IL). The results were expressed as mean and standard deviation while the distribution of genotypes and phenotypes was given as percentages. Clinical characteristics and cytokine gene polymorphisms were compared using the chi-squared test and Fisher's exact test when needed. To compare the differences between the groups, *t* test was used for independent samples. To examine whether the genotype frequencies were in Hardy-Weinberg equilibrium, goodness-of-fit *χ* ^2^-test was used. Allele frequencies were compared by a 2 × 2 contingency table using Fischer's exact test. All results were considered significant at *P* < 0.05.

## 3. Results

### 3.1. Biochemical and Epidemiological Data

The AKI patients had a lower initial renal function (calculated by MDRD equation) compared to No AKI and Control group (70 ± 51 versus 100 ± 49 and 81 ± 13; *P* < 0.001, resp.) and higher serum levels of creatinine (Table 1). AKI patients were classified as follows: 59 (41%) as injury, 25 (17%) as failure, 22 (15%) as loss, 33 (23%) as end stage, APACHE score II (20 ± 7 versus 17 ± 6; *P* = 0.001), vasoactive drug use (23 (17%) versus 5 (4%), *P* = 0.0001), mechanical ventilation (29 (21%) versus 9 (7%), *P* = 0.0001), and sepsis (44 (32%) versus 29 (21%), *P* = 0.04). Age, gender, coronary artery disease, heart failure, stroke, hypertension, and diabetes mellitus were not significantly different between the studied groups (Table 1). In the univariate analysis, only APACHE II score (O.R. 1.07 C.I. 1.04–1.10; *P* = 0.0001) and mechanical ventilation (O.R. 0.53 C.I. 0.31–0.90; *P* = 0.02) were risk factors for AKI.

### 3.2. Biochemical and Epidemiological Data according to Death

With respect to death, an association was observed between a higher APACHE II score (27 ± 7 versus 18 ± 6; *P* = 0.0001), higher serum creatinine (1.66 ± 1.49 versus 0.64 ± 0.95; *P* = 0.0001), higher CRP (13.7 ± 12.5 versus 7.1 ± 8.0; *P* = 0.001), use of vasoactive drug (42% versus 8%, *P* = 0.0001), mechanical ventilation (53% versus 11%, *P* = 0.0001), sepsis (68% versus 23%; *P* = 0.0001), and higher death rates (Table 2).

### 3.3. Genotype Polymorphisms Frequency of TGF-*β* and IFN-*γ* in Patients with and without AKI and Death

Genotype frequencies for TGF-*β* and IFN-*γ* were similar to previous reports in the Caucasian population and observed Hardy-Weinberg equilibrium.

Regarding the frequency of genotypes/alleles of the polymorphisms of the cytokines TGF-*β* and IFN-*γ*, we observed a higher prevalence expression of C/C from the TGF-*β* and T/A from the IFN-*γ* genotypes in both groups (AKI and No AKI patients) (Table 3). However, this prevalence was no significant for to develop in AKI or death.

Also, we did not observe any association between genotype profile and gender, renal function initial (measured by MDRD), specific underlying diseases, or clinical conditions occurring after ICU admission that might contribute to the course of AKI. Besides, we did not observe differences in genotypes of the TGF-*β* and IFN-*γ* polymorphisms with respect to death (Table 4) and sepsis (data not shown). We did not observe any association of haplotypes TGF-*β* and IFN-*γ* polymorphisms with AKI (Tables 5, 6, and 7).

## 4. Discussion

This is the first study aimed to investigate the prevalence of polymorphisms of TGF-*β* and IFN-*γ* in leukocytes of patients with SIRS and to investigate its possible associations as a risk factor for development of AKI and death. As expected, AKI patients had a high APACHE score II and high incidence of sepsis. However, in respect to these cytokines polymorphisms, we did not show it as risk factors to AKI or death in our population.

We observed a higher prevalence expression of genotype C/C from the TGF-*β* in both groups (AKI and No AKI patients), but this prevalence was not associated with AKI in ICU patients. The high serum levels of TGF-*β* and high TGF-*β* producer genotype have been associated with diabetic nephropathy, inflammation, and fibrosis process in chronic kidney disease [26–28]. In fact, Coll et al. described that renal fibrosis, mediated by TGF-*β*, is a common pathology implicated in all forms of kidney disease mainly in end stage renal disease (ESRD) [28]. Also, Karimi et al. reported that, in inflammatory process, the proximal tubule epithelial cell expresses high levels of TGF-*β*, which could contribute to AKI. Additionally, authors have reported in transgenic mouse model that an activation of TGF-*β* signaling in the tubular epithelium alone is sufficient to cause AKI [15].

In our study, we investigated expression of TGF-*β* polymorphisms from DNA extracted from circulating leukocytes from critically ill patients who developed AKI. AKI's patients did not show more prevalence of TGF-*β* compared to No AKI. The literature describes that patients with chronic kidney diseases have more fibrosis and it is associated with high TGF-*β* serum levels. However, the acute mechanism of this cytokine in AKI is unclear. So, to demonstrate whether TGF-*β* could have a direct effect on kidney function in patients with SIRS, it would be necessary to investigate this in renal cells from these patients.

Regarding IFN-*γ*, we observed a predominant frequency of genotype T/A in the general population. However, these genotypes were not statistically different between groups and it was not associated as a risk marker for AKI and death.

In contrast, Karimi et al. reported an association between the TT homozygote for IFN-*γ* (high producer) and acute rejection renal transplantation [29]. These authors suggest that these conditions are associated with renal injury and may contribute to evolution for AKI [7, 9]. However, in our study only 13.7% of AKI patients were high producer phenotype carriers.

Finally, our study had some limitations as follows. (1) The probability to detect polymorphisms is <1% in the overall population; therefore the inclusion of a higher number of patients should be necessary. We included 400 consecutive patients with SIRS in the ICU from a single center, where 303 were eligible for this analysis. Thus, it is possible that the sample size may have accounted for the poor correlation analysis of these polymorphisms and their associations with outcomes. (2) We investigated the main polymorphism described in the literature for each cytokine, but there are several different polymorphisms for these cytokines that could be involved in AKI pathophysiology.

Some authors have described associations between TGF-*β* and IFN-*γ* and poor prognosis such as renal fibrosis and inflammation in chronic kidney disease [30, 31]. However, there is still no data about these polymorphisms with AKI. Thus, it is possible that the measure of serum levels of these cytokines and the investigation of their polymorphisms together could have contributed to a better understanding of the molecular control and respective protein synthesis, clarifying the possible associations with outcomes.

Therefore, in the present study we concluded that the genetic polymorphisms of codons 10 T/C and 25 C/G of the TGF-*β* and +874 T/A of the IFN-*γ* were not associated as risk factors for AKI or death in our population. To validate if these studied polymorphisms may induce a renal injury in critically ill patients in ICU setting, a multicentric study with a larger sample size should be conducted.

## Figures and Tables

**Table 1:** Biochemical and epidemiological data according to the development for acute kidney injury (AKI) (*n* = 303) and healthy individuals (Control) (*n* = 244).

	AKI		Control (*n* = 244)	*P*
	Yes (*n* = 139)	No (*n* = 164)		
Age (years)	67 ± 17	65 ± 18	60 ± 10	0.67
Gender (male)	91 (66%)	97 (60%)	154 (61%)	0.26
APACHE II score	20 ± 7∗	17 ± 6	—	<0.05∗
MDRD	70 ± 51∗	100 ± 49	81 ± 13	<0.05∗
Creatinine (mg/dL)	1.07 ± 1.20∗	0.40 ± 0.70∗	1.00 ± 0.2	<0.05∗
Albumin (mg/dL)	3.06 ± 0.25	3.24 ± 0.54	4.5 ± 0.3	0.22
CRP (mg/dL)	7.89 ± 8.61	7.20 ± 8.47	0.56 ± 0.69	0.48
Vasoactive drug	24 (17%)∗	6 (4%)	—	<0.05∗
Mechanical ventilation	29 (21%)∗	11 (7%)	—	<0.05∗
Sepsis	44 (32%)∗	35 (21%)	—	0.04∗
Coronary arterial disease	26 (19%)	31 (19%)	—	0.96
Stroke	7 (5%)	13 (8%)	—	0.31
Hypertension	54 (39%)	68 (40%)	73 (29%)	0.80
Diabetes mellitus	25 (18%)	31 (19%)	32 (13%)	0.83
Chronic kidney disease	1 (0.01%)	3 (0.02%)	—	0.60
Mortality rate (%)	12 (9%)	7 (4%)	—	0.12

*AKI compared to No AKI-Student's *t* and chi-square test; ANOVA when appropriate.

**Table 2:** Biochemical and epidemiological data according to death (*n* = 303).

	Death		*P*
	Yes (*n* = 19)	No (*n* = 284)	
Age (years)	74 ± 14∗	65 ± 18	0.04∗
Gender (male)	10 (52%)	178 (62%)	0.38
APACHE II score	27 ± 7∗	18 ± 6	<0.05∗
Creatinine (mg/dL)	1.66 ± 1.49∗	0.64 ± 0.95	<0.05∗
Albumin (mg/dL)	3.00 ± 0.02	3.18 ± 0.46	0.43
CRP (mg/dL)	13.7 ± 12.5 ∗	7.1 ± 8.0	<0.05∗
Vasoactive drug	8 (42%)∗	22 (8%)	<0.05∗
Mechanical ventilation	10 (53%)∗	30 (11%)	<0.05∗
Sepsis	13 (68%)∗	66 (23%)	<0.05∗
Coronary artery disease	4 (21%)	53 (19%)	0.80
Stroke	1 (5%)	19 (7%)	0.80
Hypertension	4 (21%)	116 (41%)	0.08
Diabetes mellitus	5 (26%)	51 (18%)	0.36

*Death compared to No Death-Student's *t* and chi-square test.

**Table 3:** Frequency of allele (%) of TGF-*β* and IFN-*γ* polymorphisms in ICU patients according to acute kidney injury (AKI or No AKI) and control group.

Allele	AKI (146)	No AKI (82)	Control (98)	*P *
TGF-*β*				
Codon 10				
C/C	21 (14.3%)	11 (13.4%)	15 (15.3%)	*0.30 *
C/T	75 (51.3%)	43 (52.4%)	50 (51.1%)	
T/T	50 (34.4%)	28 (34.2%)	33 (33.6%)	
Codon 25				
C/C	3 (2%)	1 (1.2%)	3 (2%)	0.30
G/C	53 (36.3%)	30 (36.5%)	36 (36.7%)	
G/G	90 (61.7%)	51 (62.3%)	60 (61.3%)	
IFN-*γ* (+874 T/A)				
A/A	89 (36.5%)	29 (20.9%)	41 (25%)	*0.13 *
T/A	117 (48 %)	85 (61.2%)	97 (59.1%)	
T/T	34 (13.9%)	24 (17.3%)	23 (14%)	

Chi-square test and *P* < 0.05.

**Table 4:** Frequency (%) of genotypes of polymorphism from TGF-*β* and IFN-*γ* in patients according to death.

Genotype	Death		*P *
	Yes (14)	No (279)	
TGF-*β* (codon 10 e 25)			
TT GG	—	73 (26%)	n.s.
TC GG	4 (28%)	76 (27%)	n.s.
TC GC	8 (57%)	75 (26%)	n.s.
CC GG	1 (7%)	19 (6%)	n.s.
TT GC	1 (7%)	18 (6%)	n.s.
CC GC	—	13 (4%)	n.s.
CC CC	—	0 (0.7%)	n.s.
TT CC	—	3 (1%)	n.s.
IFN-*γ* (+874)			
TT	3 (21%)	43 (15%)	n.s.
TA	7 (50%)	173 (62%)	n.s.
AA	4 (28%)	63 (22%)	n.s.

Chi-square test and *P* < 0.05 or Fisher.

n.s. = not significant.

**Table 5:** Frequency (%) of haplotypes codon 10 from the TGF-*β* in patients in AKI and No AKI.

Alleles TGF-*β*	AKI (158)	No AKI (135)	*P *
Codon 10			
TT	59 (20%)	38 (12%)	0.30
TC	78 (26%)	84 (28%)	0.25
CC	21 (7%)	13 (4%)	0.48
T	197 (67%)	158 (53%)	0.50
C	121 (41%)	109 (37%)	0.50

Hardy-Weinberg equilibrium.

**Table 6:** Frequency (%) of haplotypes codon 25 from the TGF-*β* in patients in AKI and No AKI.

Alleles TGF-*β*	AKI (158)	No AKI (135)	*P *
Codon 25			
GG	95 (32%)	75 (25%)	0.30
GC	61 (21%)	55 (19%)	0.30
CC	2 (0.6%)	3 (1%)	0.25
G	253 (86%)	206 (70%)	0.20
C	65 (22%)	62 (21%)	0.20

Hardy-Weinberg equilibrium.

**Table 7:** Frequency (%) of haplotypes +874T/A from IFN-*γ* in patients in AKI and No AKI.

Alleles IFN-*γ*	AKI (158)	No AKI (135)	*P *
(+874)			
AA	42 (14%)	28 (9%)	0.65
AT	94 (32%)	84 (28%)	0.48
TT	22 (7%)	23 (7%)	0.60
A	178 (60%)	139 (47%)	0.45
T	137 (47%)	130 (44%)	0.30

Hardy-Weinberg equilibrium.
